# Plasma microRNAs are associated with acute exacerbation in idiopathic pulmonary fibrosis

**DOI:** 10.1186/s13000-016-0583-2

**Published:** 2016-11-23

**Authors:** Haiyan Min, Shanshan Fan, Shiyu Song, Yi Zhuang, Hui Li, Yongzheng Wu, Hourong Cai, Long Yi, Jinghong Dai, Qian Gao

**Affiliations:** 1 Department of Respiratory Medicine, Nanjing Drum Tower Hospital Affiliated to Medical School of Nanjing University, Nanjing, China; 2 Jiangsu Key Laboratory of Molecular Medicine, Medical School of Nanjing University, 22 Hankou Rd., Nanjing, 210093 China

**Keywords:** Idiopathic pulmonary fibrosis, Acute exacerbation, miR-25-3p, let-7d-5p

## Abstract

**Background:**

Acute exacerbation of idiopathic pulmonary fibrosis (AE-IPF) has high short-term mortality with unknown causes. To predict this malignant condition in clinics is challenging. In this study, we aim to demonstrate whether there are miRNAs that differ between AE-IPF and stable IPF, which may be served as reliable biomarker for AE-IPF prediction.

**Methods:**

Human fibrotic-associated miRNAs arrays were designed to detect miRNAs expression in plasma of 3 AE-IPF patients, 3 Stable-IPF (S-IPF) patients and 3 normal controls (NC). Differentially expressed miRNAs between AE-IPF and S-IPF patients were selected for further analyses. The validation studies were carried out in plasma of 12 AE-IPF patients, 45 S-IPF patients and 51 healthy control subjects. Signaling pathways and cellular processes interacted with validated miRNAs were predicted by DIANA-miRPath.

**Results:**

According to the array analysis, 6 miRNAs showed differentiated expression between AE-IPF and S-IPF patients (*P* < 0.05). In the validation studies, let-7d-5p was decreased in S-IPF and further decreased in AE-IPF, when compared to NC (0.0003 ± 0.0002 vs 0.003 ± 0.002, *P* < 0.01 and 0.0007 ± 0.0005 vs 0.003 ± 0.002, *P* < 0.01). While miR-25-3p was obviously decreased in S-IPF (0.0002 ± 0.0001 vs 0.0003 ± 0.0003, *P* < 0.01) but significantly increased in AE-IP (0.0023 ± 0.002 vs 0.0003 ± 0.0003, *P* < 0.01). In receiver-operator characteristic (ROC) curve analysis, the areas under the curve (AUCs) of miR-25-3p and let-7d-5p were 0.83 and 0.75, respectively. The sensitivity at fixed specificity of 90% was improved from 50% to 66.7% when the two miRNAs were combined. The functional prediction of miRNAs suggested that the loss of anti-fibrotic capacity and the gain of uncontrolled cell growth may be required in AE-IPF pathogenesis.

**Conclusions:**

In conclusion, miR-25-3p and let-7d-5p in plasma were differentially expressed between AE-IPF and S-IPF. A combination of these two miRNAs may be a potential biomarker for AE-IPF from IPF.

## Background

Idiopathic pulmonary fibrosis (IPF) is a progressive disease with steady worsening of symptoms and gas exchange. Most patients die from respiratory failure within 3 years [1–3]. Although the clinical course of IPF is usually gradual, but persistent, decline in lung function, some patients may experience an acute worsening of dyspnea and lung function with an unknown etiology [2], and is typically defined as acute exacerbations (AE) of IPF. AE-IPF may occur at any time during the disease course without an identifiable cause [1]. Approximately 50% of IPF patients died from AE [4] with a poor prognosis and a median survival of less than 3 months [5]. Current treatment strategies have consisted of high-dose corticosteroids, which has no any data from controlled trials to prove their efficacy for the patients of AE-IPF yet [5]. The pathological feature by chest high resolution computed tomography (HRCT) of AE-IPF is a diffuse alveolar damage superimposed on the usual interstitial pneumonia characteristics in IPF [6]. A better understanding of this disease is desirable.

Mature miRNAs are endogenous 18–24 nucleotide non-coding RNA molecules which act as posttranscriptional regulators of gene expression and are stable in circulation. The alterations of miRNAs in plasma or serum have been served as improved and noninvasive biomarkers for numerous pathological conditions, including cancers, autoimmune diseases and tissue injury [7–9]. Over the past years, molecular analysis of lung tissue resected for diagnostic purposes have provided more encouraging results that suggest that IPF lung biopsies have a unique mRNA transcriptome compared with non-fibrotic control biopsy samples [10, 11]. More recently, much effort has been made to define biologically relevant transcriptional differences in IPF patients with distinguishable disease progressions [10, 12]. Several studies have demonstrated that serum miRNAs were differentially expressed in IPF patients with rapidly or slowly progressive groups [13, 14]. However, it is unknown whether differentially expressed miRNAs in the circulation is associated with AE-IPF.

In this retrospective study, we initially compared the human fibrosis-related miRNAs between S-IPF and AE-IPF. Moreover, through a validation study in an expanded cohort, we determined the predictive role of the miRNAs in distinguishing between S-IPF and AE-IPF.

## Methods

### Study population

The diagnosis of IPF was recommended by the American Thoracic Society and the European Respiratory Society (ATS\ERS)[15] and was previously described [16].

The diagnosis of AE-IPF was made according to the proposed diagnostic criteria that mainly focus on a previous or concurrent diagnosis of IPF with a worsening of its clinical manifestations over 30 days or less, and the absence of infection or another identifiable etiology such as left heart failure, or pulmonary embolism [17].

A total of 63 IPF patients were admitted to Drum Tower Hospital from January 1, 2013 to June 30, 2014. Among them, 15 patients were diagnosed as AE-IPF and died within 1 month after the diagnosis. And 48 IPF patients with the stable radiographic findings and without a worsening of their clinical manifestations over a period of 30 days were defined as S-IPF. These S-IPF patients had no acute exacerbation during the follow-up period of 1–2.5 years after enrollment. In addition, 54 unrelated age-matched healthy individuals were enrolled without current or prior history of lung diseases in this study at the Center of Physical Examination of Drum Tower Hospital.

This study was approved by the Ethical Committee of the Affiliated Drum Tower Hospital of Nanjing University Medical School.

### Imaging studies

Chest HRCT was performed using various CT scanners with the patients in suspended inspiration. The criteria for inclusion of IPF in the study described previously [16]. Chest HRCT for AE-IPF generally demonstrated bilateral ground-glass abnormality with or without areas of consolidation, superimposed on the UIP pattern described previously [2]. Two radiologists, without knowledge of any of the clinical, functional, and radiographic findings, independently reviewed the HRCT of all patients.

### Plasma miRNAs preparation

Blood samples were obtained once the patients were diagnosed as S-IPF or AE-IPF. About 5 ml venous blood samples were collected in EDTA-containing tubes. Blood samples were centrifuged within 1 h in two steps. Firstly, all samples were centrifuged at 800 g for 10 min at 4 °C, and the supernatants were collected into new RNase-free tubes for a second centrifugation at 12,000 g for 10 min at 4 °C. The supernatants were then stored for RNA extraction.

Plasma total RNA including miRNAs were extracted with miRNeasy Serum/Plasma Kit (Qiagen) from 400ul plasma samples according to the manufacturer’s instructions. 100pmol/L synthesized cel-miR-39 was added into plasma as an internal calibrator to monitor extraction efficiency. The concentration and purity of the total RNA were measured by Nanodrop 2000.

### miRNAs array procedure

96-well miRNA expression profiles were used for 3 AE-IPF, 3 Stable-IPF(S-IPF) and 3 normal controls (NC). The array was designed according to miScript miRNA PCR Array (MIHS-117Z, Qiagen) including 84 human fibrosis-associated miRNAs, an external reference cel-miR-39 and an internal reference U6. First, reverse transcription (RT) of the total RNA samples was performed with TaqMan miRNA Multiplex Reverse Transcription Kit (Applied Biosystems) according to its protocol. Next, quantitative real time PCR was performed according to the protocol of TaqMan Universal PCR master mix (Applied Biosystems) on Step One plus PCR system (Applied Biosystems).

### Quantitative RT-PCR (qRT-PCR) for plasma miRNAs validation

Total miRNAs were extracted from 400 μl plasma of additional 12 AE-IPF patients, 45 S-IPF patients, as well as 51 healthy controls with PARIS Kit (Ambion), mixed with 100pmol/L synthesized cel-miR-39 as an internal calibrator to monitor extraction efficiency, and were dissolved in 50 μl elution buffer. Total miRNAs were reverse transcribed into a final volume of 30 μL cDNA with specific primers (Roche) using Prime Script RT-PCR Kit (Takara) according to the instructions. Finally, quantitative PCR was performed with UPL probe kit (Roche) using Step One plus PCR system (Applied Biosystems). The reaction conditions were: 95 °C for 10 min, and 40 cycles of 95 °C for 15 s followed by 60 °C for 1 min. Each sample was detected three times.

### MiRNAs target prediction and pathway analysis

DIANA miRPath v.2.0 was used to predict target genes by one or more miRNAs into known KEGG pathways [18]. The graphical output of the program provides an overview of the parts of the pathway modulated by validated miRNAs. The statistical significance value associated with the identified signaling pathways and biological process was calculated by the program.

### Statistical analysis

For the miRNA array and validation data analyses, the raw expression levels were determined by the cycle number at a predetermined threshold cycle (CT) <35. The fold-change for each miRNA from the control group to each patient group was calculated as 2^-∆∆CT^ with miR-16 as a reference miRNA. Relative Concentrations of miRNAs in AE-IPF, S-IPF and control plasma samples were calculated as 2^-∆CT^ and compared by the T- test. Receiver-operator characteristic (ROC) curve was generated under the nonparametric distribution assumption for expression levels of plasma miRNAs of AE-IPF and S-IPF patients by plotting sensitivity% vs (100%-specificity %). Areas under the curves (AUCs) were calculated. The biomarkers were considered to be useful when the value of AUCs were >0.7. Logistic regression was used in the combined markers analysis. All statistical analysis was performed with either SPSS17.0 or GraphPad Prism5.0 Software. 95% confidence intervals were used to quantify uncertainty, and *P* values < 0.05 were considered to be statistically significant.

## Results

### Patient characteristics

Characteristics of patients and controls including age, gender, results of lung function tests were detailed in Table 1.

**Table 1:** Characteristics of study subjects

	Array phase			Validation phase		
	S-IPF (*n* = 3)	AE-IPF (*n* = 3)	Control (*n* = 3)	S-IPF (*n* = 45)	AE-IPF (*n* = 12)	Control (*n* = 51)
Age^a^, mean ± SD	64.3 ± 13.0	63.7 ± 9.7	64.0 ± 12.0	63.9 ± 11.7	63.6 ± 12.5	65.9 ± 9.7
Female/Male	1/2	1/2	1/2	18/27	5/7	19/32
FVC^a^ (% predicted), mean ± SD	63.7 ± 17.2	NA^b^	NA	65.7 ± 10.7	NA^b^	NA
DL_CO_^a^ (% predicted), mean ± SD	56.3 ± 19.1	NA^b^	NA	51.2 ± 17.7	NA^b^	NA

*FVC* forced vital capacity, *DLCO* diffusion capacity for carbon monoxide of lung, *NA* not available

^a^Values were collected at the diagnosis. ^b^patients were unable to be tested

### Identification of pulmonary fibrosis progress-related miRNAs in plasma

**Fig. 1:**
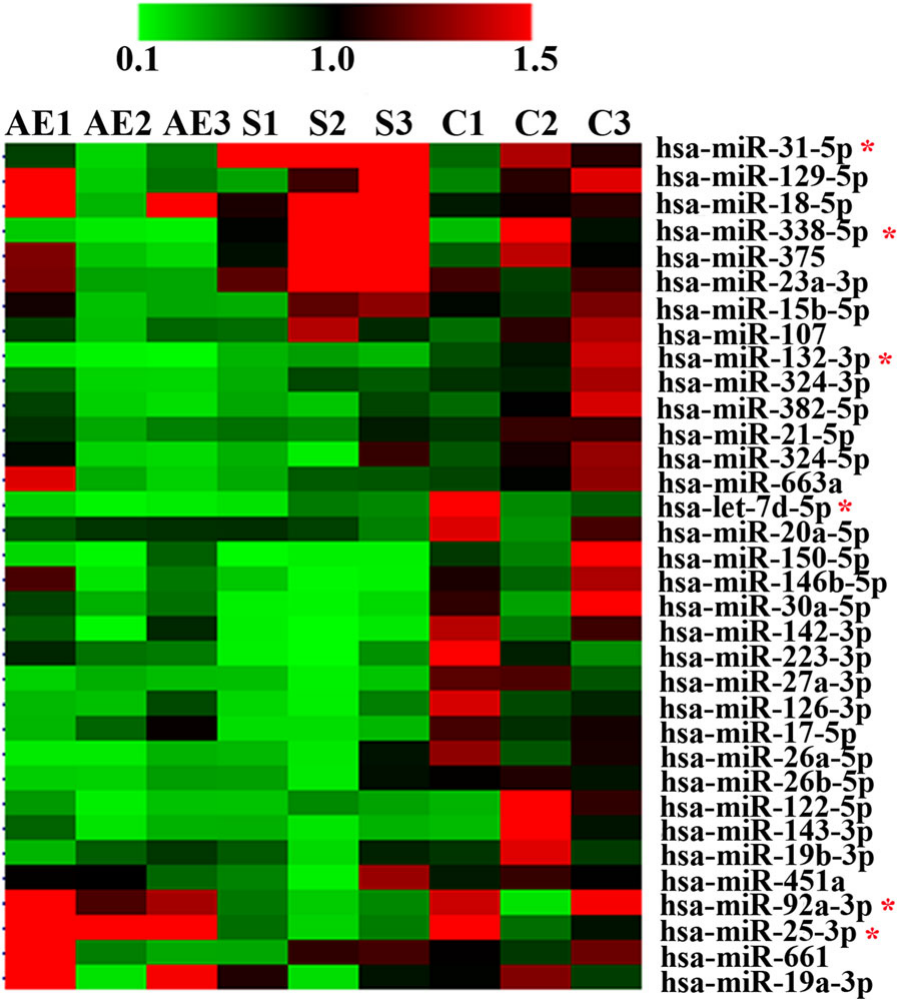
Heatmap of the expressions of miRNAs in plasma of AE-IPF, S-IPF and healthy control. Data were shown in 2^-ΔΔCT^. Red indicates miRNAs induced and green indicates miRNAs repressed. AE1, AE2, AE3 represent AE-IPF samples. S1, S2, S3 represent S-IPF samples. C1, C2, C3 represent control samples. * means *p* < 0.05

We initially profiled the expression of 84 fibrosis-related miRNAs in the plasma of 3 AE-IPF patients, 3 S-IPF patients and 3 healthy controls by the fibrosis-specific miRNAs arrays. With setting a threshold of CT < 35, a total of 34 miRNAs were further analyzed. As shown in the heat map (Fig. 1), the levels of the majority of miRNAs detected in this study were decreased in both AE-IPF and S-IPF patients, when compared to that of the controls. Among them, miR-132-3p and let-7d-5p were further decreased in AE-IPF patients when compared to that of S-IPF patients (*p* < 0.05). To identify the miRNAs that were deferentially expressed miRNAs between AE-IPF and S-IPF patients, we further compared the expressions of the miRNAs in these two fibrotic groups to the control group. We found that miR-31-5p and miR-338-5p were up-regulated in S-IPF patients (*p* < 0.05), but down-regulated in AE-IPF patients (*p* < 0.05), while miR-25-3p and miR-92-3p were up-regulated in AE-IPF group, but down-regulated in S-IPF group. All the above miRNAs were selected for further validation.

### Validation of differentially expressed miRNAs between AE-IPF and S-IPF

**Fig. 2:**
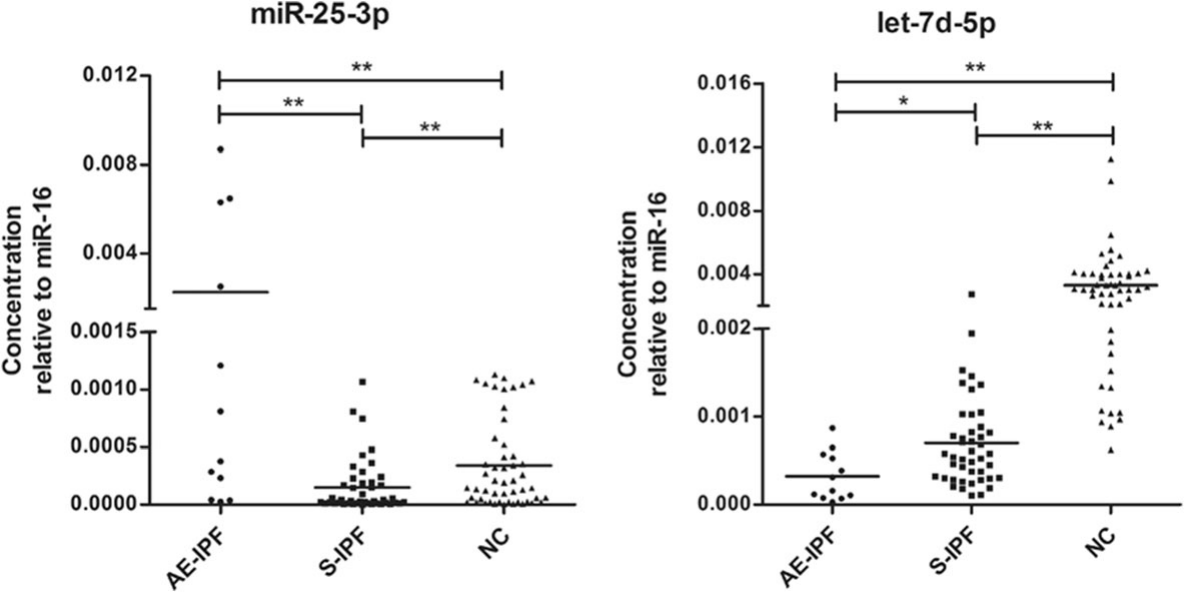
Concentration of miR-25-3p and let-7d-5p in plasma of AE-IPF, S-IPF and healthy controls. The dash presents the mean value. NC, healthy controls. * means *p* < 0.05. ** means *p* < 0.01

Concentrations of the 6 miRNAs were detected in additional 12 AE-IPF and 45 S-IPF patients, as well as 51 healthy controls by using RT-qPCR. We found that 4 of them, miR-132-3p, miR-338-5p, miR-92-3p and miR-31-5p, were not differentially expressed between AE-IPF and S-IPF groups (data not shown). As been shown in Fig. 2 and Table 2, the level of let-7d-5p was significantly decreased in AE-IPF patients, when compared to those of S-IPF patients (*p* < 0.05) and controls (*p* < 0.01). Interestingly, in the same comparison, miR-25-3p was found to be significantly decreased in S-IPF group (*p* < 0.01), but increased greatly in AE-IPF group (*p* < 0.01).

**Table 2:** Diagnostic sensitivities of measurements of single markers for predicting AE-IPF at predefined specificities

	Relative concentration to miR-16 (mean ± SD)			AUC(95% CI)	Sensitivity at 90% Specificity
	AE-IPF (*n* = 12)	S-IPF (*n* = 45)	NC (*n* = 51)		
miR-25-3p	0.0023 ± 0.0020	0.0002 ± 0.0001	0.0003 ± 0.0003	0.83 (0.70–0.96)	50%
let-7d-5p	0.0003 ± 0.0002	0.0007 ± 0.0005	0.003 ± 0.002	0.75 (0.59–0.91)	50%
miR-25-3p + let-7d-5p	—	—	—	0.83 (0.69–0.97)	66.7%

### ROC analysis

**Fig. 3:**
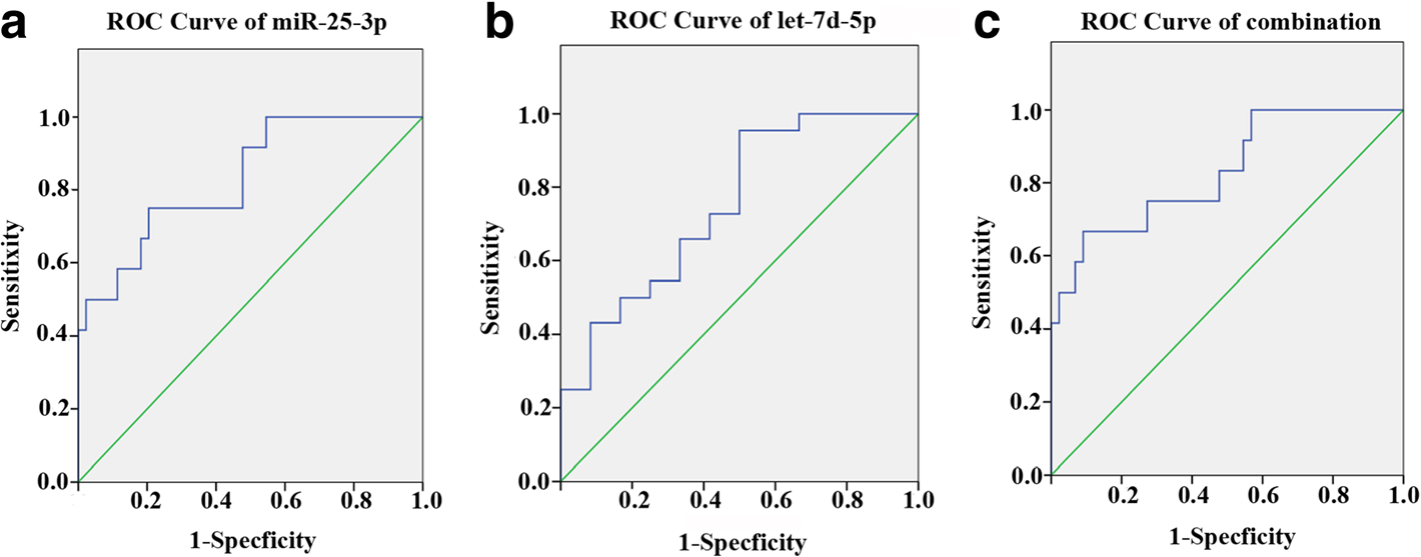
ROC curve analyses of plasma miR-25-3p and let-7d-5p as single markers and combined markers for predicting AE-IPF from IPF. **a** ROC curve of miR-25-3p as a single biomarker for predicting AE-IPF from IPF. **b** ROC curve of let-7d-5p as a single biomarker for predicting AE-IPF from IPF. **c** ROC curve of combination of miR-25-3P and let-7d-5pas a biomarker for predicting AE-IPF from IPF

To evaluate the effectiveness of the two miRNAs for predicting AE-IPF patients among IPF patients, ROC curves were constructed by comparing plasma measurements of let-7d-5p and miR-25-3p in the AE-IPF patients with that in the S-IPF patients. The diagnostic accuracy of the two miRNAs for AE-IPF prediction was calculated (Fig. 3 and Table 2). The AUCs of miR-25-3p and let-7d-5p were 0.831 and 0.752, respectively. The two candidate markers were then subject to logistic regression analysis to optimize the diagnostic utility. The sensitivity at fixed specificity of 90% was improved from 50 to 66.7%.

### Signaling pathways prediction

DIANA-mirPath analysis was applied to predict the biologic targets and pathways as well as cellular processes that miR-25-3p and let-7d-5p affected. We found that the two miRNAs shared no common direct targets at transcriptional levels. We then investigated the cellular processes interacted with miR-25-3p and let-7d-5p. We found the intersections between the two miRNAs in cell cycle related processes in an opposite way, while let-7d-5p showed the suppressive effects on these processes, miR-25-3p promoted (Table 3). Targets of miR-25-3p involved in TP53, BCL2L11 and CDKN1C, etc. The expressions of these genes were predicted to be down-regulated by miR-25-3p in AE-IPF, which suggested a proliferative potential of cells. On the other hand, the targets of let-7d-5p included TGFBR1 and CDK6. The expressions of these genes were predicted to be up-regulated in AE-IPF, which also suggested the probability of cells growth.

**Table 3:** The intersected biologic pathways of miR-25-3p and let-7d-5p predicted by KEGG pathways analysis

KEGG pathway	*p*-value	let-7-5p interacted genes	miR-25-3p interacted genes
Glioma	9.30E-06	IGF1R,KRAS,CDK6,PDGFA	TP53
Melanoma	6.97E-05	IGF1R,KRAS,CDK6,PDGFA	TP53
Chronic myeloid leukemia	0.0002533	IGF1R,KRAS,CDK6	TP53
Cell cycle	0.0017125	ESPL1,CDK6,BUB3,CDC25A	TP53,CDKN1C
Colorectal cancer	0.0034595	TGFBR1,BCL2,KRAS	TP53
PI3K-Akt signaling pathway	0.0043839	THBS1,BCL2,IGF1R,KRAS,CDK6,PDGFA	TP53,BCL2L11
Hepatitis B	0.0043839	TGFBR1,BCL2,KRAS,CDK6	TP53
Pancreatic cancer	0.0049791	IGF1R,KRAS,CDK6	TP53
Bladder cancer	0.0050961	TGFBR1,KRAS	TP53
Non-small cell lung cancer	0.0060073	KRAS,CDK6	TP53
HTLV-I infection	0.0083159	TGFBR1,KRAS,BUB3,ADCY9,PDGFA	TP53, AT2B

## Discussion

During the last decade, several studies have explored biomarkers for the prediction of rapid progressive IPF [13, 14, 19–21]. Still, AE-IPF is a less characterized condition with unidentified causes and no tools for the prediction. Current study offers an opportunity to appreciate circulating miRNAs detection as a newer standardized tool for prediction of AE-IPF.

In line with previous studies [13, 14], we found that let-7d-5p expression was decreased in the plasma of S-IPF and further decreased in AE-IPF. We were also able to show that miR-25-3p was down-regulated in S-IPF, but significantly up-regulated in AE-IPF, a new observation that indicates that S-IPF and AE-IPF are indeed separable molecularly. As a single biomarker, the AUC of miR-25-3p or let-7d-5p was 0.831 or 0.752 respectively, and the sensitivity of the combination of the two at fixed specificity of 90% was significantly improved, arguing a beneficial potential with the combination of the two miRNAs in AE-IPF determination, although the AUC value of the current combination of the miRNAs isolated in our study is still relatively low. Further studies involve a larger AE-IPF cohort and a larger panel of testing miRNAs are desirable in order to obtain an improved AUC value. Since men and women are represented in the IPF population in different proportions, we compared the levels of miR-25-3p and let-7d-5p in the plasma of female and male patients in our study in both fibrotic groups, and observed no significant differences (data not shown).

The various functional studies on the two miRNAs, miR-25-3p and let-7d-5p, should not be neglected. First, a reduction of let-7d in epithelial cells causes epithelial-mesenchymal transition and over-expression of let-7d in fibroblasts can reduce their mesenchymal properties [22, 23], suggesting that the effect of let-7d is pro-fibrotic, a process occurring in both S-IPF and AE-IPF, but significantly enhanced in latter. On the other hand, miR-25 has been found to be increased in various cancers and may act as an onco-miRNA [24–28]. The specific large increase of miR-25-3p in AE-IPF, but reduced in S-IPF, suggested that the disease may have gained a pro-growth condition. Our findings have etiological implications that acute exacerbation in IPF may be due to the collapse of anti-fibrosis mechanisms and the gain of tumorigenic molecular tendency locally or systemically. Whether the two processes are parallel or mutually interacted is not known. More importantly, whether AE-IPF may obtain a neoplastic disease-like alteration is extremely provoking, but needs cellular and pathological evidence.

Interestingly, two genes, ATP11A and OBFC1 found to be associated with IPF susceptibility in a GWAS study [29] were predicted as targets of miR-25-3p. It will be highly interesting to study the association between the two genes and miR-25-3P in IPF pathogenesis. Moreover, it is known that the shortening of telomere length is a risk factor for the survival of IPF [16, 30]. Whether one could make a link between the shortening of telomere length and the ill-expression of miRNAs in IPF progression remains to be determined.

In addition to the patients enrolled from a single medical center with a relatively small size in our study, one weakness of this investigation is that the current study was based on a scheme of one-time-point detection of the miRNAs without longitudinal measurements due to the clinical limitations, which may mask the relationship between the dynamic expressions of the miRNAs and the disease progression. Since the variability of the clinical courses of IPF are widely ranged and might be heterogeneous [31], the identification of the predictors that are capable of distinguishing the clinical courses of IPF would be a great challenge. Nevertheless, our study may have provided a clue to position miRNAs as the candidates of AE-IPF predicators.

## Conclusions

In this study, we reported that the human fibrosis-related miRNAs, miR-25-3p and let-7d-5p, were differentially expressed between S-IPF and AE-IPF. The combination of these two miRNAs was significant in predicting an acute exacerbation of IPF. The result indicates that both loss of tissue anti-fibrotic capacity and gaining of uncontrolled cell growth may be required in AE-IPF pathogenesis.

## Abbreviations

AE-IPF: Acute exacerbation of idiopathic puhlmonary fibrosis
AUCs: Areas under the curves
IPF: Idiopathic pulmonary fibrosis
miRNAs: microRNAs
NC: Normal controls
ROC: Receiver-operator characteristic
S-IPF: Stable in idiopathic pulmonary fibrosis
